# A Comprehensive Literature Review of Digital Health Interventions in the Treatment of Substance Use Disorder With Special Focus on Mobile Applications

**DOI:** 10.7759/cureus.47639

**Published:** 2023-10-25

**Authors:** Harrison R Jordan, Sidharth Sahni, Mamun M Ahmed, Joseph E Fares, Binoy V Desai, Christine N Lenchur, Richard T Jermyn

**Affiliations:** 1 Medicine, NeuroMusculoskeletal Institute, Rowan Medicine, Stratford, USA

**Keywords:** mobile technology, substance use disorder (sud), digital healthcare, drug addiction, mobile app

## Abstract

COVID-19 quarantine showed an increase in opioid-related deaths partially due to the limited capacity of clinics and treatment centers. Digital health interventions (DHIs) such as telehealth have improved access to treatment, reduced psychosocial barriers, and helped patients with substance use disorder (SUD). An in-depth literature review was conducted to gauge the efficacy and usefulness of DHIs on substance use disorder. PubMed was used with string search terms to identify studies analyzing telehealth for substance use disorders. Studies were eligible and selected if they used health interventions (HIs) and reported outcomes on the efficacy of DHIs, benefits of DHIs, and limitations of DHIs. The Agency of Healthcare Research and Quality (AHRQ) was used to analyze the impact of DHIs on SUD. Lastly, Apple’s App Store was used to identify the current DHI available. The analysis indicated that mobile phone apps were the most appropriate sources to use for patients with substance use disorders. The search also found 36 mobile applications available on the market for patients, containing mainly pain medication diaries and trackers. The study did not find any apps for clinical usage that met the standards necessary for adequate healthcare in the opioid crisis, largely due to a lack of clinician involvement in using applications. Developing adequate DHIs has the potential to improve outcomes in patients with SUD and aid in recovery time. The research concluded that physicians looking to develop DHIs should take into consideration the mode of delivery of DHI, the aim to produce specific health outcomes as opposed to multiple outcomes, and clinician involvement in DHI development. DHIs can become a vital tool for medical professionals, especially during the COVID-19 crisis, as the use of healthcare technology has limited in-person contact, maintained current doctor-patient relationships, and allowed for contact tracing of the disease.

## Introduction and background

As the prevalence of the opioid crisis continues to heavily impact many people's lives, awareness-raising efforts have been made to minimize one of the most devastating public health crises in the 21st century. The chief aim of digital health interventions (DHIs) is to reduce the adverse effects associated with the opioid endemic by utilizing technology platforms. Such interventions intend to improve the delivery and standard of healthcare in communities, institutions, and specific target populations. DHI can be used in the form of mobile phones, mobile phone apps, websites, and text messaging services. DHI has gained recent popularity in several realms of medicine as studies indicate that these interventions improve the patient's overall health [1,2]. Furthermore, the use of DHI has been pivotal in reducing the number of COVID-19 cases in various countries as evidence supports the need for these interventions to be used in contact tracing and clinical management to reduce the spread of infection [3]. While there are still many issues to resolve before it can be implemented broadly to meet the required standard of care for patients, several projects are currently in development focused on specific diseases, such as substance use disorder.

Personalized DHI options lead to increased compliance and the use of technology to improve health outcomes [4]. This emerging form of medicine has been used in numerous ways depending on the patient being treated. Still, one of the main goals of using technology to improve the healthcare system is to provide a more accessible service to the patient [4,5]. In addition, using technology in the healthcare setting enhances the ease of communication between the provider and the patient for superior long-established care [1,6]. These interventions also allow patients to be more adequately informed about their health, assess and monitor their health status, reach treatment decisions, and help titrate medicine [3].

Nicotine consumption predisposes individuals to numerous risk factors, and abstinence from smoking can significantly increase health outcomes in many patients. One of the ways DHI has improved abstinence from smoking is by providing support through text messages made explicitly for the participant, which has been influential both on its own and in combination with other interventions. At the six-month interval, researchers found that compared to the control group, the intervention group reported a higher percentage of smoking abstinence at six months (control: 4.9%, intervention: 10.7%, p<.0001). Smoking abstinence at six months was measured using a biochemically verified method. Limitations include the technique of biochemical verification. The analyzed biochemicals have a relatively short half-life; therefore, participants could have stopped smoking a few days before the testing [7]. This text to stop intervention could be used in other forms of substance abuse, such as with opioids, with similar positive results; however, future studies must develop an alternative and more accurate way to verify abstinence from substances. Furthermore, studies have shown that mobile applications geared toward smoking cessation need to address challenges in communication concerning clinical expectations and comfortability with the use of mobile app technology [7].

Studies on DHI targeting alcohol consumption show mixed results of its effects and are inconclusive if computer-based interventions can reduce alcohol consumption. While some studies suggest that computer-based interventions reduce alcohol consumption, there are many weaknesses in the research methodology, and more structured research on alcohol consumption needs to be done [8]. Nonetheless, one of the current projects in the market is the mobile application, SmarTrek, which aims to reduce alcohol consumption among college students and has been received well by participants who have tried the app. Ninety percent of them stated the SmarTrek app aided in decreasing their drinking [9]. Another moderate-quality evidence suggests that DHI can reduce alcohol consumption; however, low-quality evidence indicates no difference between DHI vs. face-to-face interventions on alcohol consumption [10].

## Review

Methodology

There were no strict criteria utilized to collect information for this literature review. However, PubMed was used with the following set of keywords: "Digital Health Interventions & Outcomes," "Digital Health Interventions & Smoking Cessation," "Digital Health Interventions & Substance Use Disorders," and "Digital Technology & COVID-19".

While gathering information about the field of DHI, we investigated various other outcomes that DHI had on substance use disorder (SUD) through the Agency of Healthcare Research and Quality (AHRQ). During our investigation, we noted the positive and negative effects that DHI had on a wide range of research participants, as well as the usefulness of the research in developing optimal interventions in the future. Furthermore, during our analysis of current interventions in SUD, we also utilized the Substance Abuse and Mental Health Services Administration (SAMHSA) to inspect and evaluate existing programs focused on the use of DHI in the management of substance use disorders. To find the current DHI in the market, we searched the Apple store for the keyword “opioid addiction.”

Results

With the opioid epidemic claiming more than 700,000 lives between 1999 and 2017 [11], substance use disorder (SUD) has become one of the biggest public health concerns in the United States. Various disciplines within the healthcare community are attempting to do their part in combating the disease, including those working on DHI [11]. Tables 1, 2, respectively, list current and future projects in the market that aim to reduce the occurrence of and support those with SUD. In addition, digital health intervention (DHI) has become increasingly involved in the SUD community. Some of the aims of DHI include enhancing physician recognition of SUD, increasing motivation for changing destructive behavior, ensuring correct usage of medication, aiding cessation of addictive substances, and many other positive changes in those suffering from substance abuse [11].

**Table 1:** Projects in Development Parameters measured by these applications include the following: mHealth implements brief motivational intervention (BMI) in an effort to increase motivation and change in behavior toward destructive substances. eQuit worRx measures smoking history, state of change, and level of dependence before supplying the patient with personalized feedback and strategies for cessation. Patient Buddy Application relays information about increased readmission risk factors from the patient and caregiver to the nurse manager and promotes early outpatient interventions while enhancing communication between the patient and clinical staff.

Projects in Development		
Project	Organization	Initiatives (Objectives/Goals)
mHealth Delivery of a Motivational Intervention to Address Heavy Drinking Among College Students	University of North Carolina, Charlotte	This project utilizes a smartphone application to assess the efficacy of and cause a change in behavior toward alcohol consumption.
Mobile app to enhance smoking cessation shared decision-making in primary care	University of Cincinnati	An initiative was developed and tested a mobile health application (eQuit worRx) with the hopes of aiding primary care providers in patient and physician decision-making with the goal of assisting in smoking cessation.
Use of Patient Buddy™ application to Disseminate Knowledge & Prevent Readmission (Virginia)	Virginia Commonwealth University	This project investigates the success of a smartphone called the Patient Buddy Application, which aims to reduce hospital admissions for patients with cirrhosis and prevent potential hepatic encephalopathy events.

**Table 2:** Digital Health Interventions in the Market DHI platforms directed toward the prevention of opioid overdose not limited to clinical settings. The software application, reSET-O, did not decrease illicit drug use or improve abstinence in patients with opioid use disorder. There are no studies that look at the long-term (>12 weeks) effects of reSET-O. DHI: digital health intervention.

Digital Health Interventions in the Market	
DHI	DHI Goals
reSET-O	reSET-O provides cognitive behavioral therapy, increases retention of patients with opioid use disorder (OUD) in outpatient treatment, and is to be used as an adjunct to outpatient treatment for patients over 18 years old who are being treated by a clinician.
Pursue care	On-demand, interventive, and transitional care to patients coming from hospital inpatient and emergency departments, primary care, and rural health clinic settings. Access to medication-assisted treatment like Suboxone from qualified physicians, addiction counseling, and mail-order pharmacy services.
FEND	Full energy, no drugs (FEND) provides access to medication-assisted treatment like Suboxone from qualified physicians, addiction counseling, and mail-order pharmacy services. FEND uses gamification, individualization, and viral content.
COR-12	Content based on current brain research, stages of change, motivational enhancement therapy, and cognitive-behavioral therapy. COR-12 offers several parameters for tracking progress, motivational support systems with daily reminders, and videos and other resources geared toward recovering opioid addict patients.
Stop OD NYC App	Stop OD NYC teaches how to recognize opioid overdose, and where to find Naloxone near you. It is geared toward preventing overdose by offering access to life-saving medication and instructs the app user on how to use the different types of Naloxone.
Buprenorphine Home Induction	Buprenorphine Home Induction Tool (BUP) is an informative mobile application that guides patients on how to conduct Buprenorphine induction using a series of questions that the patient is asked in the mobile application.

The project titled "mHealth Delivery of a Motivational Intervention to Address Heavy Drinking Among College Students (North Carolina)" specifically used a smartphone application to measure the efficacy of this type of intervention among college students who consume large amounts of alcohol [9]. Another goal of this project is to assess the feasibility and effectiveness of this type of intervention among college students who consume large amounts of alcohol. After completing two standardized surveys, 90% of the participants agreed that the specific application was easy to maneuver and even caused students to decrease their alcohol consumption [12]. Another intervention conducted at the University of Cincinnati developed and tested a mobile health app called eQuit worRx [13]. This application was created to aid primary care providers (PCPs) in distributing patient-centered outcomes research (PCOR) regarding smoking cessation and supporting shared decision-making among patients and physicians. Specifically, the application collects the smoking history, state of change, and level of dependence before supplying the patient with personalized feedback and strategies for cessation [13]. This study compared the mobile health application to the standard intervention of pamphlet distribution to patients about smoking cessation. The study found that eQuit worRx increased the time spent by patients talking about smoking cessation with their PCP and increased decision-making, even if the final decision was not to change any smoking habits [14]. Another project titled the "Use of Patient Buddy™ Application to Disseminate Knowledge & Prevent Readmission (Virginia)" uses a smartphone app called the Patient Buddy Application, which was created to reduce hospital admissions for patients with cirrhosis [15]. This program relayed information about increased risk factors from patients with cirrhosis to a nurse manager taking care of them. The study demonstrated that the application prevented numerous potential hepatic encephalopathy events. In addition, the Patient Buddy Application was found to promote early outpatient interventions and enhance communication between the patient and healthcare staff [16].

Table 2 outlines digital health interventions currently on the market online. reSET-O is a 12-week prescription digital therapeutic application intended to increase the retention rate of patients with opioid use disorder (OUD) in outpatient treatment [17]. The application provides cognitive behavioral therapy and is used as an adjunct to outpatient treatment for patients over 18 years old who are being treated by a clinician [17,18]. Another application currently on the market is "Pursue care," which assists with transitional care for patients who are not only struggling with opioid and alcohol addiction but also for those combating mental health disorders such as anxiety and depression [19]. FEND is another application that can be downloaded onto a mobile device. The acronym stands for "full energy, no drugs." This application helps explicitly identify the signs of an opioid overdose, what to do in this emergency, and even gives explicit directions on administering Narcan spray [20]. Another application with a similar purpose to REND is called "Stop OD NYC." It provides basic information about what opioids are and how they can cause life-threatening episodes in people who overdose on them. Stop OD NYC also gives directions on how to treat opioid overdose and where to find the nearest naloxone supply [21]. COR-12 is another online program that works with patients to fight opioid cravings during recovery. The application has many parts, including daily guidance and reminders, progress tracking, and a tailored relapse prevention plan [22]. Finally, the "BUP" mobile app stands for the Buprenorphine Home Induction tool and focuses explicitly on optimizing the correct medication-assisted therapy regimen. The application guides patients on how to conduct Buprenorphine induction using a series of questions that the patient is asked in the mobile application [23].

How to best use digital health interventions in substance use disorder (SUD) is unclear, especially when factoring in age. For example, one study found that Millennials (ages: 18-35) felt that social media would be the most appropriate source to implement an intervention, while Generation Z (ages: 13-17) viewed a mobile phone app as the most appropriate source to implement recovery [24]. This information should be utilized in future developments of DHI to understand the audience that would accept or resist intervention from specific digital platforms. In addition, studies have supported that digital intervention reduced alcohol consumption, while a smaller number of studies have documented no difference in alcohol consumption with DHI [8]. This lack of homogeneity about DHI in the literature suggests more research on digital health to understand its effectiveness in SUD.

Current mobile applications related to substance use disorder (SUD) focus on pain management. One study highlighted that applications in the Apple and Google Play store that included a pain diary and were in English were utilized to determine how effective these apps were for patients with pain. A total of 36 applications were found that met the criteria. Many of them included ways to keep track of the pain, such as pain location, quality, intensity, and impact of pain on the user's daily life. However, the study found that only one-third of the applications utilized clinician counsel when designing the pain diary. In addition, none of the applications included secure Health Insurance Portability and Accountability Act (HIPAA) compliance or the Pain, Enjoyment of Life and General Activity (PEG) tool used by primary care physicians for chronic pain management. It was also noted that the applications lacked cross-platform use, limiting their effectiveness. The study did not find any apps for clinical usage that met the standards necessary for adequate healthcare in the opioid crisis, primarily due to a lack of clinician involvement. Furthermore, mobile applications for substance use disorder need excellent clinician involvement and meet specific requirements to have a meaningful impact on the opioid epidemic in the United States [25].

Telemedicine use in patients with addiction is limited but has the potential to ease communication between provider and patient for more ongoing care. This analysis highlights that individuals favor videoconferencing and mobile applications when seeking online treatment for addiction. These domains of delivering healthcare supply the need for more accessible communication to the community. Some of the limitations to facilitating telemedicine found in this study include lack of reimbursement to the provider, the provider not being familiar with the technology, and confidentiality regulations. These barriers illuminated in the study need to be addressed in future mobile application technology to succeed in the digital field of medicine [6].

Developing digital health intervention tools

Digital health technology has the potential to improve healthcare delivery to patients; however, few studies are conclusive about the positive outcomes of DHI use. Therefore, it is necessary to conduct more research about DHI and its effects. In addition, researchers looking to develop digital health interventions should consider the following: 1) mode of delivery of DHI (mobile apps, social media, text messages) because the mode of delivery can target different populations (Gen Z vs. Millennials) [24]; 2) aim to produce specific health outcomes instead of multiple outcomes [4]; and 3) clinician involvement for DHI development and clinician engagement with participants. Other considerations include accurate ways to measure if specific health outcomes were achieved.

Feasibility

Through a review of the literature, there have been many benefits and limitations regarding the use of technology in healthcare. One of the enormous benefits of digital health is better communication between the patient and providers. For example, one study addressed the communication barrier between individuals in rural communities and their providers/pharmacists by allowing patients to communicate through video on their mobile application [26]. This single issue, communication with members of a rural community, illuminates the numerous possibilities for using technology in modern medicine as one of the significant barriers to healthcare is transportation. Many patients have to travel far distances to see their primary care physician, and the use of technology, in this case, a mobile application, drastically reduces the patient's transportation burden.

While there are many advantages to using digital health interventions, there are also some drawbacks. Various articles on digital health intervention have stated that the knowledge of use and comfortability with the technology is a significant impairment to the physician. Figuring out how to use different interfaces takes time away from clinicians seeing patients, which results in the provider choosing not to implement the technology fully into their practice. One of the limitations of digital health interventions is the lack of reimbursement; however, more research is needed in this area as this is a newly emerging field of medicine. Furthermore, it has been shown that training with the application/technology vastly improves its use for both the patient and the physician.

## Conclusions

Overall, DHI has emerged as an essential component in healthcare delivery as technology has allowed healthcare providers to reach a much broader patient population and enable those in rural communities to access their medical needs more efficiently. DHI has been utilized in various fields of medicine but has become increasingly important in combating the opiate epidemic in the United States and other substance use disorders as many people become introduced to substances in their youth. This aspect of SUD care delivery further supports the need for more research into the specific age groups that can be targeted with different forms of DHI, such as the preferred source of intervention through other telecommunication platforms. In closing, DHI has the potential to become a vital tool for medical professionals, especially during the COVID-19 crisis, as the use of healthcare technology has limited in-person contact, maintained current doctor-patient relationships, and allowed for efficient contact tracing of the disease.
